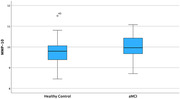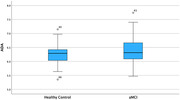# Increased levels of Matrix Metaloproteinase‐10 and Adenosine Deaminase in blood are associated with reduced cognition

**DOI:** 10.1002/alz.084942

**Published:** 2025-01-09

**Authors:** Sofia Michopoulou, Jay Amin, Christopher Kipps, Rebecca Sussams, Katie Lunnon, Clive Holmes, Jessica Teeling

**Affiliations:** ^1^ University Hospital Southampton NHS Foundation Trust, Southampton UK; ^2^ University of Southampton, Southampton UK; ^3^ University of Exeter, Exeter, Devon UK

## Abstract

**Background:**

Neuroinflammation and activation of the immune system can influence Alzheimer’s disease (AD) progression. This is mediated by inflammatory molecules, which exacerbate the production of β‐amyloid, the propagation of tau pathology, and neuronal loss. By measuring CSF markers of inflammation in a heterogeneous clinical cohort we found that levels of Adenosine Deaminase (ADA) and Matrix‐Metaloproteinase‐10 (MMP‐10) increase in patients with AD.

Despite promising results of immune markers in CSF, a substantial limitation for wide clinical application is the requirement for a lumbar puncture, which is an invasive procedure only available in specialist settings. Using alternative fluid markers, such as blood sample measurements, would enable wider use of immune markers in AD diagnosis.

Here we evaluate if measurement of ADA and MMP‐10 markers in blood samples is associated with cognitive decline.

**Method:**

Blood samples from 98 participants, including 41 healthy controls and 57 patients with amnestic mild cognitive impairment (aMCI) in the ICOS study were analysed. Participants were monitored at six‐monthly intervals over two years. Blood samples obtained during the third monitoring visit at 12 months post recruitment were analysed in this study. The levels of MMP‐10 and ADA in these samples were measured using the OLINK platform. Correlation analysis and Receiver Operator Characteristic (ROC) curves were used to evaluate if these markers of inflammation are associated with cognitive function.

**Result:**

ADA, an enzyme that regulates immune homeostasis, showed a weak but statistically significant increase in blood concentration with decreasing MoCA cognitive test scores (Rho=‐0.2, p=0.023). MMP‐10, an enzyme involved in blood brain barrier function, also showed a weak but statistically significant increase with decreasing MoCA scores (Rho=‐0.25, p=0.006).

ROC analysis indicates that both ADA and MMP‐10 are weak predictors of amnestic MCI. They can differentiate between healthy controls (MoCA 26‐30) and patients with aMCI (MoCA 18‐25) with Areas Under the Curve of only 0.57 and 0.64, respectively.

**Conclusion:**

In this study, blood levels of ADA and MMP‐10 were significantly associated with cognitive impairment. Their predictive ability of aMCI is weak and further work is required to establish the prognostic capabilities of immune markers in blood samples in larger patient cohorts.